# Performance of antigen testing for diagnosis of COVID-19: a direct comparison of a lateral flow device to nucleic acid amplification based tests

**DOI:** 10.1186/s12879-021-06524-7

**Published:** 2021-08-10

**Authors:** Maria Kahn, Lukas Schuierer, Christina Bartenschlager, Stephan Zellmer, Ramona Frey, Marie Freitag, Christine Dhillon, Margit Heier, Alanna Ebigbo, Christian Denzel, Selin Temizel, Helmut Messmann, Markus Wehler, Reinhard Hoffmann, Elisabeth Kling, Christoph Römmele

**Affiliations:** 1 III. Medical Clinic–Gastroenterology, Infectious Diseases, University Hospital of Augsburg, Stenglinstraße 2, 86156 Augsburg, Germany; 2 Laboratory Medicine and Microbiology, University Hospital of Augsburg, Stenglinstraße 2, 86156 Augsburg, Germany; 3 Chair of Health Care Operations/Health Information Management, University of Augsburg, Universitätsstraße 16, 86159 Augsburg, Germany; 4 COVID-19 Task Force, University Hospital of Augsburg, Stenglinstraße 2, 86156 Augsburg, Germany; 5 General and Special Pathology, Faculty of Medicine, University of Augsburg, Stenglinstraße 2, 86156 Augsburg, Germany; 6 IV. Medical Clinic-Emergency Department, University Hospital of Augsburg, Stenglinstraße 2, 86156 Augsburg, Germany; 7 Department of Hygiene and Environmental Medicine, University Hospital of Augsburg, Stenglinstraße 2, 86156 Augsburg, Germany

**Keywords:** (3–10): SARS-CoV-2, COVID-19 testing, SARS-COV-2 antigen testing, Point-of-care testing

## Abstract

**Objectives:**

The gold standard for diagnosing an infection with SARS-CoV-2 is detection of viral RNA by nucleic acid amplification techniques. Test capacities, however, are limited. Therefore, numerous easy-to-use rapid antigen tests based on lateral flow technology have been developed. Manufacturer-reported performance data seem convincing, but real-world data are missing.

**Methods:**

We retrospectively analysed all prospectively collected antigen tests results performed between 23.06.2020 and 26.11.2020, generated by non-laboratory personnel at the point-of-care from oro- or nasopharyngeal swab samples at the University Hospital Augsburg and compared them to concomitantly (within 24 h.) generated results from molecular tests.

**Results:**

For a total of 3630 antigen tests, 3110 NAAT results were available. Overall, sensitivity, specificity, NPV and PPV of antigen testing were 59.4%, 99.0%, 98.7% and 64.8%, respectively. Sensitivity and PPV were lower in asymptomatic patients (47.6% and 44.4%, respectively) and only slightly higher in patients with clinical symptoms (66.7% and 85.0%, respectively). Some samples with very low Ct-values (minimum Ct 13) were not detected by antigen testing. 31 false positive results occurred. ROC curve analysis showed that reducing the COI cut-off from 1, as suggested by the manufacturer, to 0.9 is optimal, albeit with an AUC of only 0.66.

**Conclusion:**

In real life, performance of lateral-flow-based antigen tests are well below the manufacturer's specifications, irrespective of patient’s symptoms. Their use for detection of individual patients infected with SARS-CoV2 should be discouraged. This does not preclude their usefulness in large-scale screening programs to reduce transmission events on a population-wide scale.

**Supplementary Information:**

The online version contains supplementary material available at 10.1186/s12879-021-06524-7.

## Introduction

The initial local outbreak of the "coronavirus disease 2019" (COVID-19) in Wuhan in December 2019 has become a worldwide pandemic [1]. One of the main reasons for the successful spread of the virus is the infectiousness before the onset of symptoms [2]. Transmission occurs mainly via droplet infection [3].

The gold standard for the diagnosis of an acute SARS-CoV2 infection is the direct pathogen-specific detection of the RNA of SARS-CoV2 via nucleic acid amplification tests (NAAT) in samples from the respiratory tract [4]. However, due to the worldwide spread of COVID-19 and the resulting high demand for test reagents, availability is limited. Moreover, NAAT are often carried out in batches and take 6–8 h, resulting significant delays until results are reported. This has a huge impact on many processes in hospitals.

As an alternative to NAATs, numerous (> 200, as of 01.12.2020) point-of-care antigen test (AgPOCT) for direct virus detection are currently available [5, 6]. As opposed to NAATs, these tests detect viral protein antigens by immunological means in naso- or oropharyngeal swabs. Similar to NAATs, test performance is hugely influenced by preanalytical factors like the quality of the swab itself or the sample extraction. These tests, however, are easy to perform, do in theory not require highly trained staff or specialized laboratory equipment and yield results in only a couple of minutes.

The virus detection limit (LOD = Limit of Detection: copies / ml or TCID50/ml) of rapid antigen tests is significantly higher (by several powers of ten) compared to molecular genetic testing methods (e.g. NAAT) [7]. Reliable statements on the effects of the increased LOD of antigen tests on diagnostic sensitivity and specificity or predictive value in clinical practice are lacking, especially in a real-life context at the point-of-care, where the tests are performed by patient’s caretakers rather than by highly trained and specialized laboratory staff [7, 8].

Therefore, we evaluated the real-life performance of the SARS-CoV2-AgPOCT between June and November 2020 at the point-of care in our third level university hospital and compared its performance to simultaneously performed NAATs.

## Methods

The study is a monocentric retrospective analysis of prospectively collected data at Augsburg University Hospital. The Standard F Covid19 Ag FIA / SD (Biosensor) detects SARS-CoV-2 nucleoprotein in a swab sample by means of lateral immunochromatography with fluorescence-detection on a dedicated reader (model F2400 or F. 200, Bestbion). A Cut-off-Index (COI) is calculated as the ratio between test and control bands, and a COI ≥ 1 is reported and labelled “positive”. For comparison, the following NAATs were performed in the molecular biology department: cobas SARS-CoV2 (Roche), Aptima SARS-CoV2 assay (Hologic), GeneXpert SARS-CoV2 (Cepheid), RealStar SARS-CoV2-RT-PCR kit (Altona). All tests were performed according to the manufacturer’s recommendations, on specialized equipment (Roche cobas 6800, Hologic Panther).

### Study population

At Augsburg University Hospital, AgPOCT have been used decentrally at various locations since 23.06.2020. All documented antigen tests were recorded for the study between 23.06.2020 and 26.11.2020. Indications for antigen testing (in addition to universally performed NAAT screening) included: absence of symptoms suggestive of COVID-19, admission from other hospitals or nursing homes, urgent interventions or diagnostic procedures with an anticipated need for admission to intensive or intermediate care units. All NAAT results were generated within 24 h of antigen testing.

### Test performance

Swabs for AgPOCT and NAAT were usually taken simultaneously, usually oropharyngeal swabs. NAATs were performed in the Department of Molecular Medicine at the Institute for Laboratory Medicine and Microbiology. Results were recorded in the laboratory information management system (Swisslab, Nexus), data were extracted by SQL queries. For the AgPOCT, the following parameters were manually recorded: results (positive/negative), COI value, patient’s symptoms (symptomatic/asymptomatic), simultaneous PCR swab (yes/no).

### Ethics

The study has been approved by the Ethics Committee of the Ludwig-Maximilians-University Munich (reference number: 20-1052). The patient consents to the data collection within the framework of this study by signing the treatment contract with the University Hospital Augsburg. The performance of the examinations used complies with the specifications of the Declaration of Helsinki.

## Results

### Entire cohort

Between June 23, 2020 and November 26, 2020, a total of 3630 antigen tests were performed, 3110 had a concomitant NAAT result. Of these, 96 samples (3.1%) were positive by NAAT. 57 (59.4%) of the NAAT-positive samples were also positive by antigen test (Additional file 1).

Thus, diagnostic sensitivity and specificity of the AgPOCT were 59.4% and 99.0%, respectively, compared to NAAT. Given a calculated prevalence of 3% in our study population, negative and positive predictive values were 98.7% and 64.8%, respectively (Table 1).

**Table 1:** Point-of-Care test results of AgPOCT compared to NAAT of samples tested within 24 h

SARS-CoV2	Entire cohort			Asymptomatic patients			Symptomatic patients		
	Ag positive	Ag negative		Ag positive	Ag negative		Ag positive	Ag negative	
NAAT positive	57	39		20	22		34	17	
NAAT negative	31	2983		25	2273		6	192	
Sensitivity (%)			59.4			47.6			66.7
Specificity (%)			99.0			98.9			97.0
PPV (%)			64.8			44.4			85.0
NPV (%)			98.7			99.0			91.9

### Symptomatic vs. asymptomatic patients

Among 2589 tests performed in the emergency department, 249 (9.6%) were from patients with clinical symptoms consistent with COVID-19 disease (Table 1).

In symptomatic patients, sensitivity of antigen tests compared to NAATs is higher than in asymptomatic patients (66.7% vs. 47.6%). The calculated prevalence was 16% and 1.9% in symptomatic and asymptomatic patients, respectively. This results in a PPV of 85% in Ag-positive symptomatic patients compared to 44.4% in asymptomatic controls. Consistently, diagnostic specificity (98.9% and 97.0%) and negative predictive values (99.0% and 91.9%) are lower in symptomatic patients than in asymptomatic controls (Table 1).

### COI values

Cut-off indices (COI) are calculated as the ratio between test and control bands on the lateral immunochromatography test and can therefore be considered a measure of signal strength. COI values were available for 2460 of the 3110 (79.1%) AgPOCT with concomitantly generated NAAT results. Table 2 shows COI values in different groups compared to NAAT testing. Notably, COIs in samples which are NAAT positive are significantly higher than in samples that are positive by Ag testing but negative by NAAT (Ag-testing false positive).

**Table 2:** Documented COI values measured with fluorescent immunoassay readers analyzing the antigen test

COI	Number	Mean	Minimum	Maximum	95%-confidence intervall
Total	2460	0.76	0	58.16	[0.57; 0.94]
NAAT positive	75	19.22	0	58.16	[14.84; 23.62]
NAAT negative	2237	0.17	0	5.80	[0.16; 0.19]
Ag false positive	27	1.67	1.00	5.80	[1.25; 2.09]
Ag false negative	25	0.22	0	0.96	[0.12; 0.32]

**Fig. 1:**
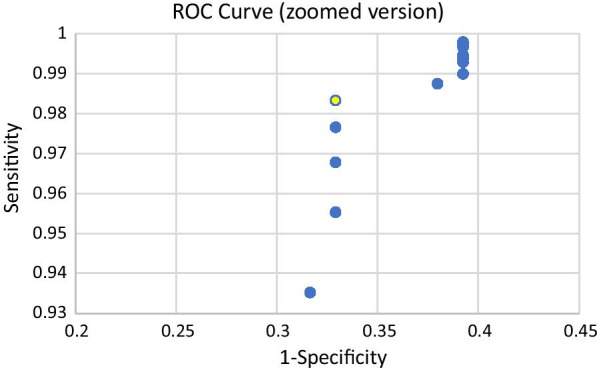
ROC curve for sensitivity and specificity for different COI thresholds. The highlighted point marks the optimal value with a COI threshold value of 0.9

Considering the low sensitivities achieved with AgPOCT, we evaluated whether test performance could be improved by optimizing the COI threshold for calling a test result “positive”. We generated a ROC analysis, evaluating COI cut-offs between 0.5 and 2.5 in steps of 0.1. Compared to the manufacturer’s recommendation of COI = 1 as threshold, we find improved performance when lowering the COI threshold to 0.9 (Fig. 1).

This results in an AUC of 0.66 (compared to 0.61 with COI cut-off of 1). Sensitivity, specificity, PPV and NPV in the entire cohort were 62.5%, 98.7%, 60.0% and 98.8%, respectively, and hence slightly improved with a COI cut-off of 0.9 compared to 1.0.

### Unspecifically reactive antigen test results

In 27 of 31 (87.1%) samples with positive results in AgPOCT which were negative by NAAT, a COI value was documented. In these samples, the COI was significantly lower than in samples positive by both NAAT and AgPOCT, suggesting that these samples are unspecifically reactive (Table 2, compare rows 2 and 4). To exclude false-negative NAAT results, an average of 3.4 (range, 1–13) NAAT assays were performed; all of which remained negative. Three patients in whom only a single NAAT was performed were in good clinical condition and were therefore discharged home to the care of the health authorities. Occasionally, we noted that blood contained in the swab material leads to a false positive Ag testing result. We retrospectively analysed all patient data and found that 10 of the 31 (32.2%) patients with unspecifically reactive Ag test results had evidence of bleeding in the mouth and throat. The COI values of blood-contaminated samples did not differ significantly from those of uncontaminated samples (Table 3).

**Table 3:** Evaluation of Ct values of patients tested positive or false negative by antigen test

Ct values	Number	Mean	Minimum	Maximum	95%-confidence intervall
Total	74	26.5	13.0	42.7	[25.1; 28.0]
Ag correct positive	46	25.1	15.7	37.0	[23.6; 26.5]
Ag false negative	28	29.0	13.0	42.7	[26.1; 31.8]

### False-negative antigen test results

In the entire cohort, the antigen test remained negative despite positive NAAT result in 39 patients. For 28 (71.8%) of these cases, Ct-values were available for analysis. These did not differ between Ag correct positive and Ag false negative samples (Table 3). Table 4 shows that the sensitivity of Ag testing improved by 5% if only clinically relevant samples with Ct values < 30 were considered positive. The de-isolation of patients with CT values > 30 is common practice in German health authorities based on the recommendations of Robert-Koch-Institute.

**Table 4:** Performance of antigen test (AgPOCT) according to RT-PCR Ct values

SARS-CoV2	Ct < 30 as clinically relevant			Sensitivity (%) of different Ct cut-offs			
	Ag positive	Ag negative		Ct < 25	Ct 25 to < 30	Ct 30 to < 35	Ct > 35
Ct value < 30	52	29		74.2	69.2	30.0	33.3
Ct value > 30 negative	36	2993					
Sensitivity (%)			64.2				
Specificity (%)			98.8				
PPV (%)			59.1				
NPV (%)			99.0				

### Sensitivity depending on the Ct values

Finally, we wished to analyse whether AgPOCT performed better in highly positive samples with low Ct values in PCR tests. We therefore analysed sensitivity of Ag testing in groups of samples generated by different Ct value cut-offs (< 25, 25–30, 30–35, > 35). Even in the group of highly positive samples (Ct < 25), sensitivity of Ag testing reached only 74%, with 8 of 31 samples negative by Ag testing. Sensitivity of Ag testing further decreased with increasing Ct values, reaching only 33% in low-positive samples by PCR (Table 4).

## Discussion

The currently ongoing COVID-19 pandemic generates a substantial need for testing patients and other groups for active virus replication and, presumably, infectiousness. Local, state or federal authorities require patients and/or health care personnel to be tested upon hospital admission or on a regular basis. Other applications include testing of visitors before entry into nursing homes or visiting public venues. Traditionally, these tests are performed by NAAT, e.g. RT-PCR or transcription-mediated amplification. However, NAAT-test resources are limited and a routinely performed NAAT-test requires several hours, professional laboratory equipment as well as laboratory skilled personal. Frequent und ubiquitous testing for SARS-CoV-2 poses one of the central elements of the German nationwide pandemic response—e.g. for school children or working environments without options for home office. In this context, the evaluation of various test systems and the implications of their (negative) results in terms of reliability, quality and usability is essential and has to take the above presented findings including limitations into account.

Thus, there is a clear need to increase testing capacity with easy-to-perform tests that can be done by low-trained personal, do not require specialised equipment and provide quick results. One solution could be the increased use of antigen tests based on lateral flow immunochromatography and a large number of tests have been introduced in the market in recent months. Manufacturer-reported test characteristics are favourable: the German Federal Institute for Drugs and Medical Devices lists 312 tests with sensitivities of > 80% and specificities > 97% as compared to PCR [6]. An independent comparative evaluation is underway under the auspices of the Paul-Ehrlich-Institute [9]. In collaboration with WHO, the Foundation of Innovation for New Diagnostics (FIND) summarises data from independent evaluations of in-vitro diagnostics and diagnostic performance data including rapid point-of-care COVID-19 antigen tests [10].

However, we could not confirm these favourable test characteristics in clinical practice. Analysing > 3000 samples with concurrently generated NAAT-results, which is by far the largest series to date, we find a low overall sensitivity of < 60%. Sensitivity was even lower (< 50%) in asymptomatic persons. Even in patients with suspected high viral loads (as determined by PCR testing with a Ct < 25), the test sensitivity of 74% is far from being optimal—or even in the manufacturer-reported range. This is well in line with earlier, much smaller series: Liotti et al. [11] report an overall sensitivity of 47%, and Paul et al. [12] report an overalls sensitivity of 71%, with slightly better results (sensitivity 86%) in symptomatic cases, but even worse performance (sensitivity 39%) in asymptomatic patients. Furthermore, FIND published clinical data from Brazil (n = 453) and Germany (n = 676) with equal or better test performance results (clinical sensitivity: 69.2% and 77.5%; clinical specificity: 96,9% and 97,9% respectively). For Ct-values under 25 the sensitivity even improved up to 87,9% and 100% [13]. These differences between the performance stated by the manufacturer and the actual performance are due to several factors. Firstly, manufacturer-reported data are generated on a well characterized sample set for which the results are known, and the test is performed by highly trained personnel, who often have been involved in test development. These personnel know how to read the tests and are well aware of potential pitfalls, e.g. how to interpret weak bands or other forms of ambiguous results. Secondly, pre-analytical factors are much less a concern in controlled laboratory settings than under real-life conditions. For example, in the busy and often turbulent environment of a large emergency department, the procedure of taking nasopharyngeal swabs may be performed differently by different people. Hence, the quality of the sample may vary widely. This may be much less a concern in highly sensitive tests like NAAT, who will probably give positive test results even in suboptimally generated samples. It may, however, seriously impact on results of less sensitive antigen tests. Of course, one may argue that medical staff should be obliged to respect preanalytical standards and perform sample collection as required—but as we all know, this is hardly possible under certain circumstances.

One may argue that antigen testing may be used to exclude inapparent SARS-CoV2 infection, since—even in our hands—the negative predictive values are quite high (99% in asymptomatic patients). However, this depends on the impact of overlooked positive test results. In situations where it is necessary to identify every possibly infectious person, e.g. before invasive endoscopic procedures, in order to protect medical personnel or other patients—the impact of even a single overlooked infectious patient is substantial, so the high negative predictive values do not translate into increased clinical security.

Therefore, in clinical and highly sensitive environments (e.g. nursing homes) the testing strategy for SARS-CoV-2 should not be solely based on antigen testing. A robust, risk-adapted concept is needed to define indications for the faster and easier antigen-test or timely and in terms of resources more demanding NAAT-testing. In a clinical environment the combination of both concepts can facilitate the daily routine. For example, patients with negative Ag-testing can be transferred to a non-COVID ward but should be separated until the NAAT-result is available. Obviously, antigen-testing cannot replace standard hygiene precautions like distancing, hand hygiene or using face masks. However, one has to keep in mind that it is currently unclear how test positivity (by any test) translates into clinical infectiousness. It has been shown that infectiousness of samples in cell culture decreases with decreasing viral loads measured by PCR [14]. Even for these data, it is not clear how they translate into clinical infectiousness and person-to-person spread.

Outside a clinical or highly sensitive environment on the other hand, antigen testing could be extremely useful if it is used to reduce the number of transmission events in a population. Mina et al. [15] recently suggested a testing strategy in which an entire population is screened with high frequency. This maximises the chance that patients are tested in the phase of high viral loads, where antigen tests have maximum sensitivity. Infected patients can then quickly be isolated and further transmissions from these patients can be prevented. Importantly, it does not matter if individual patients are overlooked—even when only half of the transmission chains are identified and index patients are isolated from the population, this effectively reduces transmission in the population as a whole and may be sufficient to reduce the reproduction rate to < 1, eventually leading to elimination of the virus from the population. Of course, the more sensitive the tests are and the more frequent they are performed, the quicker can virus elimination from the population be achieved.

There are clear limitations to our study. While the number of performed AgPOCT and NAAT is quite large, the number of CT-values available is limited. Another limitation is that different swabs were used for performing AgPOCT and NAAT. This may have resulted in varying swab quality between the individual samples. As we have only examined one AgPOCT based on lateral flow immunochromatography from a specific manufacturer, no statement can be made about other tests. Further studies are needed to evaluate different tests under realistic clinical conditions.

As mentioned above we noted that blood contained in the swab material leads to a false positive Ag testing result. It cannot be precluded that any other contamination of the swab material may also lead to false positive test results. However, through the Paul-Ehrlich-Institute, minimum criteria for SARS-CoV-2 antigen tests have been made for their use under the German government's coronavirus test regulation. These minimum criteria include, among others, the validation of tests with regard to cross-reactions with related coronaviruses and interferences with e.g. influenza viruses or Staphylococcus aureus [16].

From a clinical point of view, however, false negative test results are more problematic, as they could lead to infectious persons not being isolated[17]. Not only poor swab quality can lead to false negative results. The significance of the test result also depends on patient factors, such as the time since the onset of the disease, the immune status and the quantity of the viral load or of the specific target protein expression, e.g. nucleocapsid versus spike proteins [5]. A study by Hirotsu et. al. showed that antigen tests based on chemiluminescence enzyme immunoassay only produce reliable results for moderate to high viral loads [18].

In situation where individual patient results are of interest (e.g. screening of a patient on admission to a hospital, clarification of a clinical suspicion), the diagnostic strategy for COVID-19 disease should not be based solely on rapid antigen testing due to the low analytical and clinical sensitivity. A rapid point of care antigen test has many advantages; one of them is the fast availability of the test result within 20 to 30 min. However, there is a not inconsiderable risk of an overlooked infection with the risk of an uncontrolled outbreak of infection in a highly vulnerable health care institution, especially in times with a high prevalence of disease. Then risk far exceeds all the advantages of a rapid antigen test.

Since a negative test result does not rule out an infection (the infectivity may be reduced), the risk of misinterpreting a negative test result can simulate a false sense of security.

This does not preclude their usefulness in large-scale screening programmes to reduce transmission events across the population.

## Conclusion

This study was able to show that the real-life performance of the examined lateral-flow-based antigen test was significantly below the manufacturer's specifications. This indicates that the use of this test for the detection of individual patients infected with SARS-CoV2 should be discouraged.

Particularly in vulnerable situations, e.g. screening patients in hospital or to exclude infections with SARS-CoV2 in case of relevant symptoms, the diagnostic strategy for COVID-19 disease should not be based exclusively on rapid antigen tests due to the low analytical and clinical sensitivity. There is a non-negligible risk of overlooked infection with the danger of an uncontrolled outbreak of infection in a high-risk healthcare facility.

## Supplementary Information

**Additional file 1.** Emergency department workflow for antigen testing.

## Data Availability

The datasets generated and analysed during the current study are available from the corresponding author on reasonable request.

## Abbreviations

Ag: Antigen test
AgPOCT: Point-of-care antigen test
AUC: Area under the curve
COI: Cut-off-index
COVID-19: Corona virus disease 2019
Ct: Cycle threshold
FIA: Fluorescence immunoassay
FIND: Foundation of Innovation for New Diagnostics
LOD: Limit of detection
NAAT: Nucleic acid amplification test
PCR: Polymerase chain reaction
RT-PCR: Reverse transcription polymerase chain reaction
SARS-CoV2: Severe acute respiratory syndrome coronavirus type 2
