# Supplementary material for: Performance of antigen testing for diagnosis of COVID-19: a direct comparison of a lateral flow device to nucleic acid amplification based tests
Source: BMC Infect Dis. 2021 Aug 10;21:798. doi: 10.1186/s12879-021-06524-7 (PMC8354301; doi:10.1186/s12879-021-06524-7)
Supplement: Supplementary file 1 — Additional file 1. Emergency department workflow for antigen testing. [file 12879_2021_6524_MOESM1_ESM.docx]

**Cover sheet**

Original article

Performance of antigen testing for diagnosis of COVID-19 – a direct comparison of a lateral flow device to nucleic acid amplification based tests

Maria Kahn^1^*, Lukas Schuierer^2^*, Christina Bartenschlager^3^, Stephan Zellmer^1^, Ramona Frey^3^, Marie Freitag^3^, Christine Dhillon^4^, Margit Heier^1^, Alanna Ebigbo^1^, Christian Denzel^5^, Selin Temizel^6^, Helmut Messmann^1^, Markus Wehler^5^, Reinhard Hoffmann^2^, Elisabeth Kling^2^, Christoph Römmele^1^

^1^: III. Medical Clinic – Gastroenterology, Infectious Diseases, University Hospital of Augsburg, Stenglinstraße 2, 86156 Augsburg, Germany
^2^: Laboratory medicine and microbiology, University Hospital of Augsburg,
Stenglinstraße 2, 86156 Augsburg, Germany
^3^: University Center of Health Sciences at University Hospital of Augsburg (UNIKA-T), Chair of Health Care Operations/Health Information Management, Faculty of Business and Economics, University of Augsburg, Universitätsstraße 16, 86159 Augsburg, Germany
^4^: General and special pathology, Faculty of Medicine, University of Augsburg,

Stenglinstraße 2, 86156 Augsburg, Germany
^5^: IV. Medical Clinic - Emergency Department, University Hospital of Augsburg,
Stenglinstraße 2, 86156 Augsburg, Germany
^6^: Department of Hygiene and Environmental Medicine, University Hospital of Augsburg,
Stenglinstraße 2, 86156 Augsburg, Germany

* shared first authorship

**Corresponding author:**

Maria Kahn
III. Medical Clinic –

Gastroenterology, Infectious Diseases

University Hospital of Augsburg
Stenglinstraße 2, 86156 Augsburg
Maria.Kahn@uk-augsburg.de
0821/400-165397


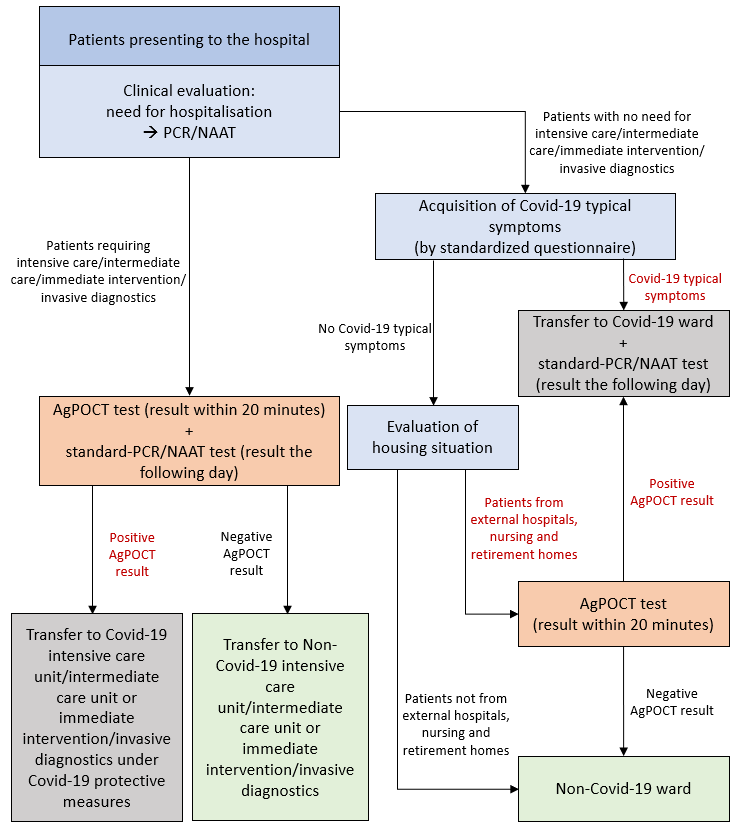
*.*
